# Noncovalently Immobilized Glucose Oxidase/Horseradish Peroxidase Cascade on Polyamide Supports for Eco-Friendly Polyaniline Synthesis

**DOI:** 10.3390/molecules30143003

**Published:** 2025-07-17

**Authors:** Nadya V. Dencheva, Joana F. Braz, Sofia A. Guimarães, Zlatan Z. Denchev

**Affiliations:** IPC—Institute for Polymers and Composites, University of Minho, 4800-056 Guimarães, Portugal; joanabraz@dep.uminho.pt (J.F.B.); a101413@alunos.uminho.pt (S.A.G.)

**Keywords:** enzyme-mediated polymerization, glucose oxidase/horseradish peroxidase cascade, enzyme immobilization, polyamide microparticles, polyaniline, green polymer chemistry

## Abstract

This study discloses the noncovalent immobilization of a bienzyme cascade composed of glucose oxidase (GOx) and horseradish peroxidase (HRP) onto magnetically responsive polyamide microparticles (PA MPs). Porous PA6, PA4, and PA12 MPs containing iron fillers were synthesized via activated anionic ring-opening polymerization in suspension, alongside neat PA6 MPs used as a reference. Four hybrid catalytic systems (GOx/HRP@PA) were prepared through sequential adsorption of HRP and GOx onto the various PA MP supports. The initial morphologies of the supports and the hybrid biocatalysts were characterized by SEM, followed by evaluation of the catalytic performance using a two-step glucose oxidation cascade process. Among all systems, the GOx/HRP@PA4-Fe complex exhibited the highest activity, being approximately 1.5 times greater than the native enzyme dyad, followed by the PA6-supported system with slightly inferior performance. All systems obeyed Michaelis–Menten kinetics, with the immobilized cascades displaying higher *Kₘ* and *Vₘₐₓ* values than the non-immobilized enzyme pair while maintaining comparable catalytic efficiencies, *CE* (*CE* = *k_cat_*/*Kₘ*). Subsequently, the immobilized and native enzyme systems were employed for the polymerization of aniline. According to UV–VIS, complete monomer conversion was achieved within 24 h for selected catalysts, and FTIR analysis confirmed the formation of polyaniline in the emeraldine base form without the use of template molecules. These findings highlight the potential of Fe-containing polyamide microparticles as efficient supports for the sustainable, enzyme-mediated synthesis of intrinsically conductive aromatic polymers.

## 1. Introduction

In recent years, a growing industrial practice has involved the use of isolated enzymes derived from living organisms as catalysts for polymer syntheses in vitro. The hallmark of enzyme catalysis is its superior catalytic activity and high selectivity under mild reaction conditions, typically at ambient temperatures and most often in aqueous media, while avoiding toxic reagents [1]. Consequently, enzyme-mediated polymerizations are considered the most natural and sustainable means to synthesize polymers, many of which are intended for biomedical applications such as surgical glues or dental fillings, where biocompatibility is crucial [2]. Since the early 2000s, the concept of Green Polymer Chemistry has emerged as a main approach for achieving a sustainable society. The development of novel enzyme-mediated synthetic pathways, even for conventional polymers, is a central part of this concept [3,4].

Among the numerous enzymes studied as polymerization biocatalysts, oxidoreductases have been identified as particularly suitable for radical polymerizations, especially of vinyl and substituted aromatic monomers [5]. Oxidoreductases can be divided into oxidases and peroxidases, depending on whether they use oxygen or hydrogen peroxide as a substrate. The most commonly utilized enzymes for radical-mediated polymerization include laccase, glucose oxidase (GOx), and horseradish peroxidase (HRP), used individually or in combination [2,6,7,8,9]. These enzymes can generate radical species either directly or through a secondary reaction involving an enzyme-derived product, eliminating the need for thermal or photo initiators typically required for free radical polymerizations. This makes one- or bienzyme systems based on oxidoreductases (the latter also known as enzyme cascades) attractive candidates for sustainable radical polymerization of commercial monomers.

Polyaniline (PANI) was one of the first intrinsically conductive polymers to gain industrial significance following the discovery of its electrical conductivity [10,11]. Today, PANI finds application across a broad spectrum of fields, including drug delivery systems, photovoltaic devices, rechargeable batteries, display technologies, microelectronics, and chemical sensors [12].

Conventional synthesis of PANI typically involves chemical or electrochemical oxidation of aniline (ANI), but these methods require harsh conditions such as extreme pH, strong oxidants, and toxic solvents, raising sustainability concerns [13]. In contrast, enzymatic routes employing oxidoreductases offer greener alternatives. Laccase (O_2_-dependent) [14,15] and HRP (H_2_O_2_-dependent) [16,17,18] are commonly used for PANI synthesis, but HRP is inclined to inactivation at high peroxide concentrations. To address this, Kurisu et al. [19] developed a bienzyme cascade system using native GOx/HRP and D-glucose to generate H_2_O_2_ in situ, minimizing peroxide-induced deactivation during the ANI polymerization.

Despite their advantages, native enzymes—either single or in cascades—often suffer from poor stability and limited reusability [20]. Immobilization enhances enzyme stability under stress conditions (e.g., excessive pH, temperature, organic solvents) and simplifies catalyst recovery, especially when magnetic supports are used [21,22,23]. Moreover, co-immobilization often enhances the performance of enzyme cascades through mechanisms such as substrate channeling [24,25,26] or modification of the local microenvironment [27,28,29].

Our previous studies demonstrated that microparticulate polyamide 6 (PA6 MP), a structural analogue of proteins, can noncovalently interact with enzymes via hydrogen bonding and serve as an effective support for immobilizing enzyme cascades, with potential biosensing applications [30,31]. In the present work, we synthesized several polyamide microparticles—PA4, PA6, and PA12—with and without Fe fillers to confer magnetic sensitivity. GOx and HRP were sequentially absorption-immobilized onto each support to form hybrid catalytic systems (GOx/HRP@PA). These were compared to the GOx/HRP cascade in terms of immobilization efficiency, catalytic activity, and kinetic parameters. All immobilized systems were then applied, for the first time, to catalyze the radical polymerization of ANI in the absence of any template molecules, demonstrating the feasibility of sustainable enzymatic synthesis of conductive polymers using noncovalently immobilized enzyme cascades.

## 2. Results

### 2.1. Synthesis of PA MPs and Their Morphology by SEM

Four types of polyamide (PA) MP supports were synthesized via activated anionic ring-opening polymerization (AAROP) in suspension, as described in Section 3.2. The PA6 MP support without Fe appeared as a white powder, whereas all other Fe-containing supports were gray and exhibited ferromagnetic behavior. The most important characteristics of the four empty PA MPs are summarized in Table 1. The chemical structures of the monomers, catalysts, and resulting polymers are schematically presented in Figure S1 of the Supporting Materials.

As shown in Table 1, the AAROP in suspension produced purified PA MPs in good yields, ranging from 56% to ~60% based on the lactam monomer. The content of methanol-extractable oligomers varied from 2 to 5 wt.%, with the highest found in the PA4-Fe sample. The viscosity average molecular weights (*M_η_*) were in the range of 25–30 kDa, except for PA4-Fe, which had a lower value of 18 kDa. This value should be interpreted with caution, as it was estimated using the Mark–Houwink parameters *K* and *α* for PA6 due to the absence of specific data for PA4 in H_2_SO_4_.

**Figure 1:**
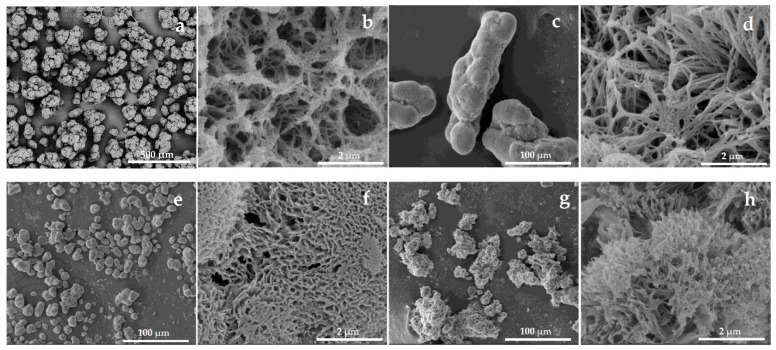
SEM micrographs at different magnifications of empty as-prepared PA MP supports: (**a**,**b**) PA6; (**c**,**d**) PA6-Fe; (**e**,**f**) PA4-Fe; (**g**,**h**) PA12-Fe. For sample designation, see Table 1.

The SEM images depicted in Figure 1 revealed notable differences in particle shape, size, and surface morphology. PA6 MPs (Figure 1a) were the largest (100–200 µm), consisting of aggregates of nearly spherical subunits measuring 25–40 µm. The PA6-Fe sample (Figure 1c) showed a broader size distribution (30–150 µm) but also featured high-aspect-ratio, pea-pod-like aggregates, most probably formed by the magnetic alignment of Fe particles followed by encapsulation with in situ-formed PA6. PA4-Fe MPs were the smallest, with oval shapes and sizes in the 10–30 µm range (Figure 1e). PA12-Fe MPs formed irregular aggregates ranging from 20 to 100 µm (Figure 1g).

Surface topographies obtained at higher magnification also varied substantially. PA6 and PA6-Fe microparticles (Figure 1b,d) displayed mesh-like morphologies, with cavity diameters in the range of 100–1000 nm. In PA6-Fe, the distinct surface structure is likely due to the presence of Fe, which may act as a nucleating agent during cold crystallization at the final stage of AAROP. PA4-Fe and PA12-Fe samples, also observed at high magnification (Figure 1f,h), exhibited sponge-like textures, with well-defined pores dominating over closed cavities. The average pore sizes were 120–300 nm for PA4-Fe and 200–800 nm for PA12-Fe. These differences are most likely attributable to the distinct AAROP processing temperatures: 40 °C for PA4-Fe and 95 °C for PA12-Fe, which influence crystallization behavior and the resulting morphology.

It should be noted that the sizes of the GOx macromolecule (~7.0 nm × 5.5 nm × 8.0 nm, ~160 kDa) and HRP (~4.0 nm × 5.0 nm × 7.0 nm, ~40 kDa) are significantly smaller than the pore dimensions of all the supports, permitting the expected sequential stacking and confinement of the two enzymes upon immobilization.

In the conclusion of the SEM study of neat supports, the four PA MP types exhibit markedly different sizes and surface morphologies, allowing for a systematic investigation of how these parameters affect the performance of the immobilized GOx/HRP enzyme cascades.

### 2.2. Noncovalent Immobilization of GOx/HRP on PA MPs

**Figure 2:**
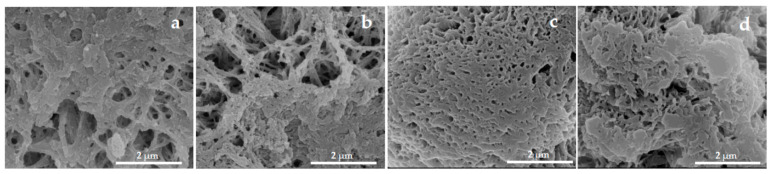
Close-up SEM micrographs of MP supports with immobilized GOx/HRP: (**a**) PA6; (**b**) PA6-Fe; (**c**) PA4-Fe; (**d**) PA12-Fe.

The sequential adsorption-based immobilization of the bienzyme cascade onto the PA MPs—first HRP, followed by GOx—resulted in four hybrid catalytic systems, generically designated as GOx/HRP@PA, where PA = PA6, PA6-Fe, PA4-Fe, or PA12-Fe. As shown in the close-up SEM micrographs in Figure 2, both enzymes are deposited on the surfaces of the PA MPs, leading to the visible obstruction of surface pores and cavities. Previous studies on GOx/HRP immobilization onto PA6 microparticulate supports, investigated via synchrotron X-ray scattering, demonstrated that this process induces denser packing of the protein matrices of the apoenzymes. This densification arises from extensive hydrogen bonding at the enzyme–support interface and is accompanied by the filling of surface and subsurface cavities [30,31].

**Figure 3:**
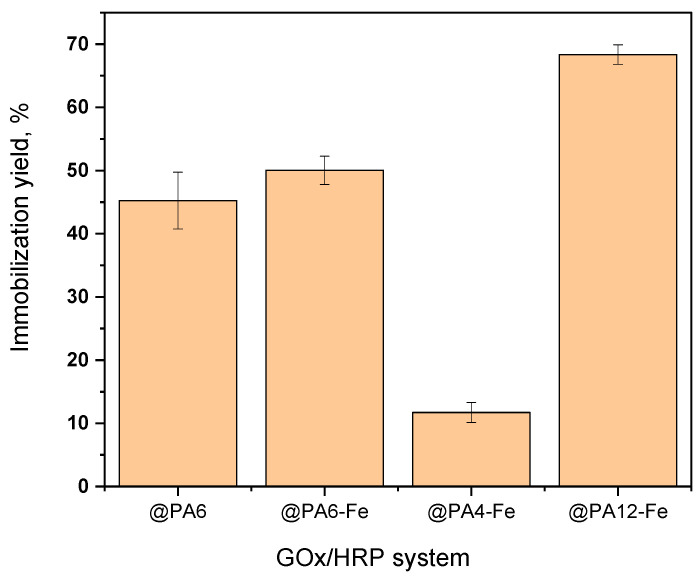
Immobilization yield of GOx + HRP on different PA MP carriers. Percentages refer to the total theoretical protein amount. Results represent the average of three independent immobilization experiments and illustrate the influence of MP porosity and morphology on the amount of enzymes immobilized.

The immobilization yield of total protein (GOx + HRP) on each support was determined as a percentage of the theoretical amounts contained in the HRP and GOx solutions used during immobilization and is presented in Figure 3. Based on calculations using Equation (2), the amount of immobilized protein on each PA microparticle correlates strongly with the porosity of the respective carrier. PA12-Fe MPs, which display the largest pores and a sponge-like morphology, retained up to 68% of the total theoretical protein introduced during sequential HRP and GOx immobilizations. In contrast, PA4-Fe, with the smallest particle size and finest pore structure, immobilized only about 12%. The PA6 and PA6-Fe supports, characterized by intermediate porosity and mesh-like surface structures, achieved immobilization yields of approximately 45–50% of the available enzymes.

As shown in our previous studies on the GOx/HRP cascade [30,31], only proper sequential immobilization (first HRP, then GOx) on the same PA MPs ensures the optimal catalytic performance of this cascade. Reversing the order or using a premixed enzymes solution for random co-immobilization consistently led to significantly lower activity. These results strongly suggest a spatial distribution where GOx is predominantly located near the PA MP surface, while HRP is retained deeper within the porous structure.

### 2.3. Comparative Activity Studies

The overall catalytic activities of the four immobilized GOx/HRP@PA systems were evaluated and compared to that of the GOx/HRP system. For this purpose, the consecutive bienzymatic cascade reaction illustrated in Figure S2 of the Supplementary Materials was employed. This reaction mechanism was first described by Josephy et al. [32] and later confirmed by Misono et al. [33]. In the first step, glucose is oxidized by the molecular oxygen present in the reaction medium under the catalytic action of GOx, yielding gluconic acid and H_2_O_2_. In the second step, HRP uses the generated H_2_O_2_ to oxidize the 3,3′,5,5′-tetramethylbenzidine (TMB) chromogenic substrate, forming the cation-radical species TMB•^+^. This radical subsequently participates in the formation of a blue–green charge–transfer complex [TMB···DTMB], which is detectable by UV–VIS spectroscopy at 652 nm. Notably, if either of the two enzymatic reactions in Figure S2 does not occur, or if the sequence of reactions is disrupted, the final chromogenic product (i.e., the TMB charge–transfer complex) will not be formed, and no change in absorbance at λ = 652 nm will be observed.

**Figure 4:**
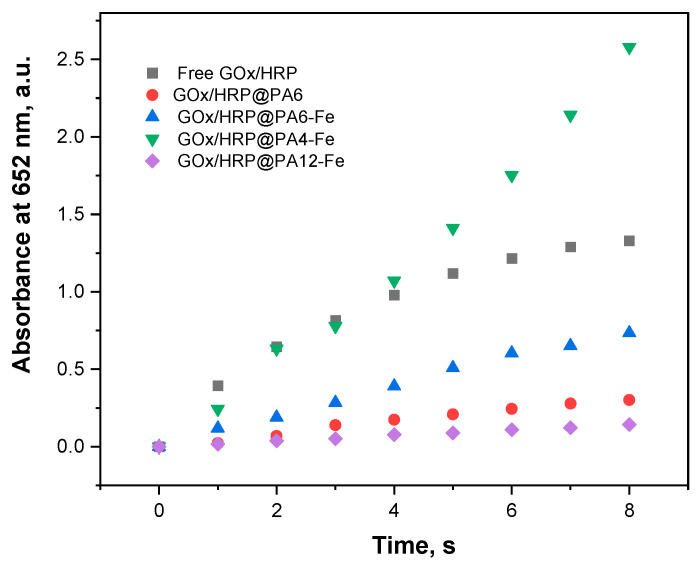
Overall catalytic activities (room temperature, 0.1 M acetate buffer, pH = 4.5) of all GOx/HRP@PA immobilized systems compared to those of the native enzyme dyad. For details, see Section 3.6.

The catalytic activity of each bienzyme system was therefore assessed by monitoring the time-dependent increase in absorbance at 652 nm, as shown in Figure 4. The most important activity parameters are presented in Table 2. To enable accurate comparison, the total protein content in each hybrid GOx/HRP@PA and the GOx/HRP system (Figure 3) were used to normalize the slope of the linear absorbance–time curves. Specific activities were thus calculated in μkat·L^−1^ per mg protein content. The final column in Table 2 summarizes the relative activities, with the activity of the GOx/HRP system set as 100%.

Figure 4 and the data in Table 2 show that the catalytic activities based on the initial reaction rates of the GOx/HRP and the GOx/HRP@PA4-Fe complexes are nearly identical during the first 4–5 s of the reaction. Notably, the relative activity of the PA4-Fe immobilized cascade is approximately 50% higher than that of the native enzyme system. In contrast, the other three immobilized systems—GOx/HRP@PA6, -PA6-Fe, and -PA12-Fe—exhibit significantly lower relative activities, ranging from 10% to 55% of the native enzymes benchmark. A general inverse trend is observed between the amount of immobilized protein and the catalytic activity: the higher the total protein content immobilized on the support, the lower the observed activity parameters. This suggests that the chemical nature and morphology of the polyamide support can play a critical role in defining enzyme performance.

Our choice of the HRP:GOx molar ratio was based on the comprehensive study by Mackey et al. [34], which evaluated cascade activity across a range from 1:2 to 26:1 under identical glucose concentrations. The authors observed the best performance at a 1:1 molar ratio, corresponding in our case to 0.005 mg/mL HRP and 0.015 mg/mL GOx (Section 3.6).

The activity of the GOx/HRP@PA system should be related to the spatial arrangement of the enzymes within the porous support. In all systems, the enzymes are immobilized sequentially, first HRP and then GOx. This procedure should result in a stratified distribution within the support: HRP tends to localize deeper within the pores, while GOx remains closer to the surface. The two enzymes likely occupy distinct yet adjacent domains, where differences in the microstructure and the proximity of these domains will be expected to influence the efficiency of intermediate diffusion (O_2_ and H_2_O_2_) between active sites.

The exceptional performance of the PA4-Fe support, which immobilizes the lowest total amount of protein, may be due to the formation of thinner enzyme domains within the smaller pores on the MP surface, minimizing diffusion distances and facilitating more effective substrate and intermediate transport. Additionally, the PA4 matrix offers a higher density of hydrogen bonding interactions between the –NH–C(O)– moieties of the polymer chains and the immobilized enzymes, most probably contributing to a more favorable orientation of their active centers.

In summary, the architecture of enzyme co-immobilization and the chemical structure of the support, particularly the density of amide groups along the polymer backbone, have a decisive impact on the catalytic efficiency of the GOx/HRP cascade. This principle appears to have general validity for engineered multi-enzyme biocatalysts, as previously suggested by Lin et al. [35].

### 2.4. Comparative Kinetic Studies

**Figure 5:**
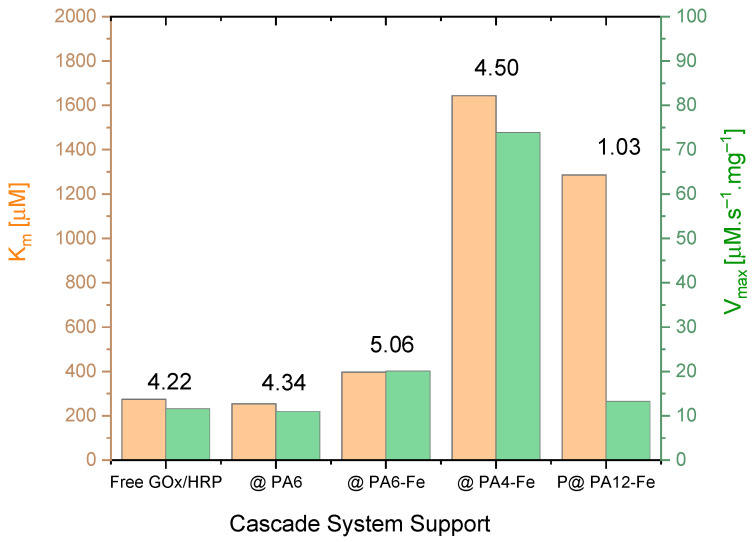
Direct comparison of the kinetic parameters *Vₘₐₓ* (normalized to total protein content, thus becoming equal to *k_cat_*) and *K_m_* for the native GOx/HRP cascade and the four systems immobilized on PA MP supports. The numerical values above the bars represent the catalytic efficiency (*CE* × 10^2^) in s^−1^. For clarity, only the support designations are shown on the *X*-axis. See the main text for additional details.

The effect of the different PA MP supports on the kinetics of the GOx/HRP-catalyzed cascade reaction was investigated by varying the concentration of glucose (the first substrate) while maintaining a constant concentration of TMB (the second substrate), as detailed in Section 3.7. The initial reaction rates *V*_0_ were determined in the 0–30 s interval. Michaelis–Menten saturation curves were then constructed by plotting *V*_0_ against the glucose concentration. For more accurate determination of the kinetic parameters, double reciprocal Lineweaver–Burk plots according to Equation (3) were generated (see Figure S3a–e in the Supplementary Materials). From these plots, the Michaelis–Menten constant (*K_m_*) and the maximum reaction velocity (*Vₘₐₓ*) normalized for the total protein amount were derived and are presented in Table 3 and Figure 5.

This simplified approach to kinetic analysis of the GOx/HRP cascade in the presence of the TMB chromogen has been widely applied in previous studies [36,37]. The method provides a useful general overview of the cascade kinetics, as the formation rate of the final chromogenic product depends on several interconnected factors: the glucose concentration; the amount and diffusion rate of in situ generated H_2_O_2_; and its ability to reach the active site of HRP, producing thereafter a colored product measurable over time. While this approach does not capture the full mechanistic complexity of the bienzyme system, it provides practical benchmarks for comparing the catalytic performance of different GOx/HRP@PA systems. Naturally, such conclusions are only valid under strictly controlled experimental conditions (i.e., constant pH, enzyme loading, temperature, and substrate concentrations) that were strictly maintained throughout this study.

Notably, the above simplified approach is consistent with the predictions made in the pioneering theoretical work by Hemker and Hemker [38], who proposed that, under certain circumstances, multi-enzyme cascades may exhibit kinetic profiles that resemble those of single-enzyme catalysis.

Analysis of the kinetic data in Table 3 and Figure 5 reveals several important trends. First, the *Vₘₐₓ* values normalized by the total enzyme concentration correspond, in general, to the turnover number *k_cat_* (see Table 3, column 3). Notably, the highest *k_cat_* value is observed for GOx/HRP@PA4-Fe, followed by the cascade immobilized on PA6-Fe, while the remaining systems exhibit similar *k_cat_* values in the range of 12–13 µmol·s^−1^.mg^−1^.

For all biocatalysts, *k_cat_* values represent only a small fraction of the corresponding *K_m_* values (typically between 1% and 5%), indicating that *K_m_* in this case primarily reflects the enzyme–substrate binding affinity. From Table 3, it can be inferred that the GOx/HRP@PA4-Fe system exhibits relatively low substrate affinity, although coupled with the highest *k_cat_* value. In contrast, the GOx/HRP@PA12-Fe system shows both low affinity and low catalytic turnover, whereas the cascades immobilized on PA6-based supports display *K_m_* values comparable to those of the native enzyme system.

The *k_cat_*/*K_m_* ratio, also known as the catalytic efficiency (CE) (Table 3, last column), combines information about the substrate binding and catalytic rate and is particularly useful for comparing enzyme performances under substrate-limiting conditions. As observed, with the exception of the GOx/HRP@PA12-Fe system that shows a notably low CE, the remaining immobilized cascades exhibit CE values comparable to that of the native enzyme system. The GOx/HRP@PA6-Fe and GOx/HRP@PA4-Fe complexes stand out, displaying the highest catalytic efficiency among the immobilized GOx/HRP cascades.

These results suggest that most all PA MP-supported cascades could be promising for use as effective biocatalysts. Among them, the native enzyme system, along with the PA6-Fe- and PA4-Fe-supported cascades, is expected to perform best at low-to-moderate substrate concentrations due to its relatively low *K_m_* values. However, at high substrate concentrations, the GOx/HRP@PA4-Fe system is likely to outperform all others, benefiting from its significantly higher *k_cat_* value.

### 2.5. Oxidative Polymerization of ANI by GOx/HRP Cascades

#### 2.5.1. Reaction Scheme

**Figure 6:**
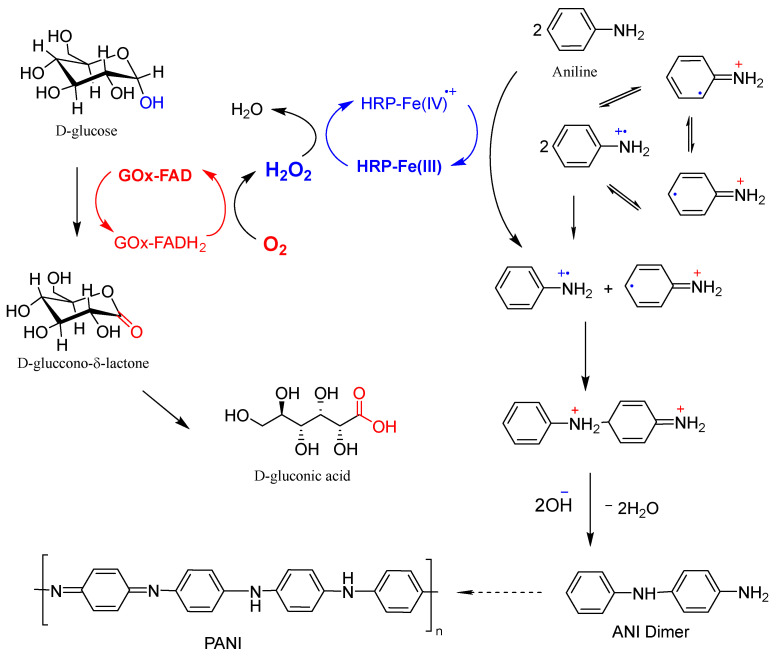
Schematic representation of the GOx/HRP cascade reaction used for the oxidation of ANI monomers to anilino radical-cations, which subsequently undergo coupling reactions, leading to oligomer and polymer formation (adapted from [19]). For further details, see the main text.

The generally accepted scheme for the enzymatic polymerization of ANI using the GOx/HRP bienzyme cascade with native enzymes for PANI synthesis is presented in Figure 6. The redox-active FAD coenzyme of GOx oxidizes D-glucose to glucono-δ-lactone, which transforms easily into D-gluconic acid while itself being reduced to FADH_2_. Molecular oxygen dissolved in the reaction medium then re-oxidizes FADH_2_ back to FAD, producing H_2_O_2_ in situ, which is subsequently reduced by HRP. Thereby, the Fe^3+^ heme center of HRP is oxidized to a high-valent Fe^4+^=O moiety, forming a porphyrin π-cation-radical. This intermediate catalyzes two sequential one-electron oxidations of ANI molecules, generating ANI radical-cations and regenerating the resting Fe^3+^ state of HRP.

The two ANI radical-cations undergo coupling reactions with neutral aniline and/or with each other, leading to the formation of oligomeric and polymeric products. These propagation steps most likely proceed without further direct enzymatic involvement [17]. The role of template molecules that are often added in enzymatic PANI syntheses, using native enzymes to control the polymer growth direction and enhance its structural order, is not depicted in Figure 6 but is well documented in the literature [16,17,18,19].

The enzymatic polymerization of ANI thus follows the same biocatalytic oxidation logic as the TMB transformation illustrated in Figure S2, proceeding through a similar cation-radical intermediate. However, while TMB oxidation typically halts after one or two electron transfers, resulting in a stable, colored product, the oxidation of ANI continues through multiple coupling steps. These subsequent reactions lead to the formation of linear or branched PANI structures, depending on the specific reaction conditions.

When the PA MP-immobilized GOx/HRP systems developed in this study are used for ANI polymerization, colored water-soluble oligomers form immediately after the reaction is triggered by the addition of glucose (see Section 3.8). Notably, no template molecules are required in this process, as the high-molecular-weight PANI, being insoluble in the aqueous medium, is deposited directly onto the PA MP supports. This results in a visible color change in the microparticles from white or light grey to black. It is hypothesized that the deposited PANI layer interacts strongly with the polyamide macromolecules through hydrogen bonding.

**Figure 7:**
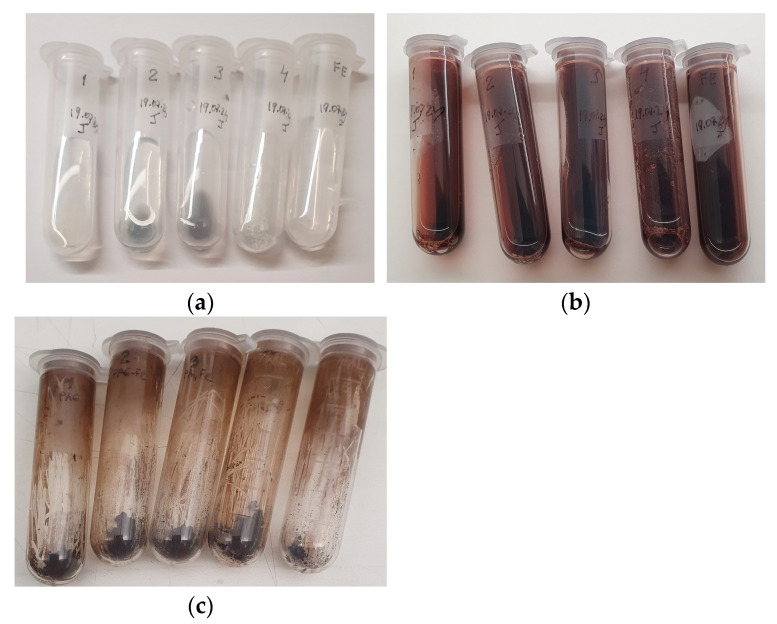
Visible color changes in the reaction media for all studied ANI polymerizations employing GOx/HRP cascade: (**a**) initial state before starting of polymerization; (**b**) after 240 min of polymerization; (**c**) after 1440 min (24 h) of polymerization and natural evaporation of the aqueous solvent. 1—GOx/HRP@PA6; 2—GOx/HRP@PA6-Fe; 3—GOx/HRP@PA4-Fe; 4—GOx/HRP@PA12-Fe; FE—native (free) GOx/HRP.

The visible color changes observed in the reaction media during enzyme-mediated ANI polymerization using all catalytic systems investigated are presented in Figure 7. Before glucose is added to initiate the reaction (Figure 7a), all reaction media are colorless and transparent, clearly revealing the white or grey PA MP supports. In the early and intermediate stages of the polymerization (Figure 7b), the color of both the solution and the microparticles transitions through various shades of dark red and brown. After 24 h and the natural evaporation of water, all samples display a uniformly black appearance (Figure 7c), indicating extensive PANI formation and deposition upon the PA MPs.

#### 2.5.2. UV–VIS Investigation of the Water-Soluble Fractions

**Figure 8:**
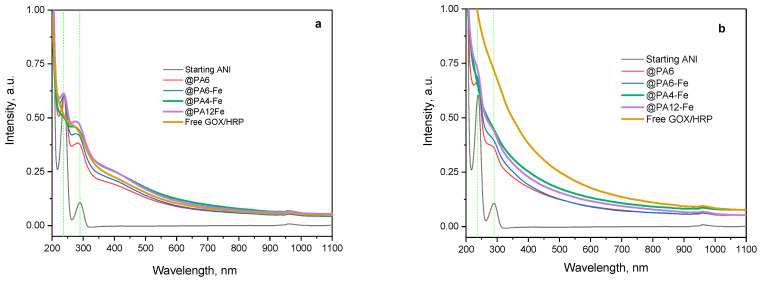
UV–VIS determination of the concentration of ANI monomer during its enzymatic polymerization to PANI catalyzed by the native GOx/HRP cascade and by PA MP-immobilized bienzyme systems. (**a**) After 90 min of reaction; (**b**) After 240 min of reaction. The UV–Vis spectra were recorded in acetonitrile, as described in [39]. The green dashed vertical lines indicate the positions of the two characteristic peaks of the initial ANI monomer.

Following this qualitative color analysis, the evolution of the ANI concentration in the reaction mixture was quantitatively monitored over time using all catalytic systems investigated: the non-immobilized GOx/HRP cascade and the four MP-immobilized variants (Figure 8). UV–VIS spectra in acetonitrile were recorded as previously described [39] to quantify the amount of unreacted aniline monomer after 90 and 240 min of reaction. This method is based on the formation of stable complexes between acetonitrile and aniline, which exhibit two distinct absorbance bands around 230 nm and 290 nm, as shown in Figure 8a,b.

As the polymerization progresses during the first 90 min (Figure 8a), the concentration of ANI decreases, which is reflected by a reduction in resolution between the two absorbance bands and their gradual attenuation. Among all systems, the GOx/HRP@PA4-Fe complex and the native enzyme dyad show the most pronounced decline of the ANI bands at 90 min, in agreement with the kinetic data presented in the previous section.

After 4 h of polymerization (Figure 8b), residual ANI absorbances are still detected with the GOx/HRP@PA6 and GOx/HRP@PA6-Fe hybrid biocatalysts, whereas the two ANI bands are completely absent with the other catalytic systems, indicating near-complete monomer conversion. It is important to note that neither high-molecular-weight PANI nor the PA MP supports are soluble in acetonitrile, confirming that the spectral changes observed correspond solely to the remaining unreacted ANI in the reaction media.

**Figure 9:**
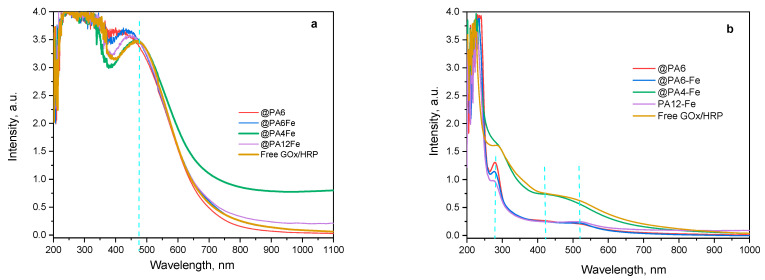
UV–VIS determination of the water-soluble PANI during the enzymatic polymerization of ANI catalyzed by the GOx/HRP and by the PA MP-immobilized cascades. (**a**) After 90 min of reaction; (**b**) After 240 min of reaction. The blue dashed vertical lines indicate the positions of characteristic peaks in the reaction mixture.

Additional structural information about the forming PANI can be obtained from the UV–VIS spectra of the water-soluble oligomeric fractions produced as the polymerization of ANI progresses (Figure 9).

During the first 90 min of reaction (Figure 9a), all samples exhibit strong absorbance in the 450–465 nm region, corresponding to π–π* transitions of the benzenoid segments characteristic of the emeraldine base form of PANI [40]. Notably, no peaks associated with the fully oxidized form of PANI, typically observed in the 700–900 nm region, are present at this stage of the reaction.

After 240 min of reaction time (Figure 9b), the higher-molecular-weight fractions of PANI have precipitated from the aqueous medium. Residual absorbance at 290 nm, corresponding to unreacted ANI monomer, is still observed in the systems catalyzed by GOx/HRP@PA6, GOx/HRP@PA6-Fe, and GOx/HRP@PA12-Fe. In contrast, for both the native enzyme cascade and the PA4-Fe-supported system, only absorbance signals in the 400–500 nm range (most likely arising from water-soluble PANI oligomers) are detected.

These findings once again confirm the superior catalytic efficiency of the GOx/HRP enzyme system and the GOx/HRP@PA4-Fe biocatalyst. Additionally, in any of the studied systems, no absorbance bands are observed above 700 nm, which is typically associated with delocalized polarons or fully oxidized PANI fractions, e.g., pernigraniline base.

#### 2.5.3. Characterization of the Water-Insoluble PANI Fractions

Given the extensive literature on the synthesis and structure of neat PANI and its composites [41], it is well established that the PANI repeat units can comprise alternating amine and imine groups, commonly referred to as benzenoid and quinoid rings. The relative content of these units determines the three principal oxidation states of PANI, as schematically illustrated in Figure S4 of the Supplementary Materials. Notably, only the emeraldine base can be transformed into the electrically conductive emeraldine salt through protonic doping.

**Figure 10:**
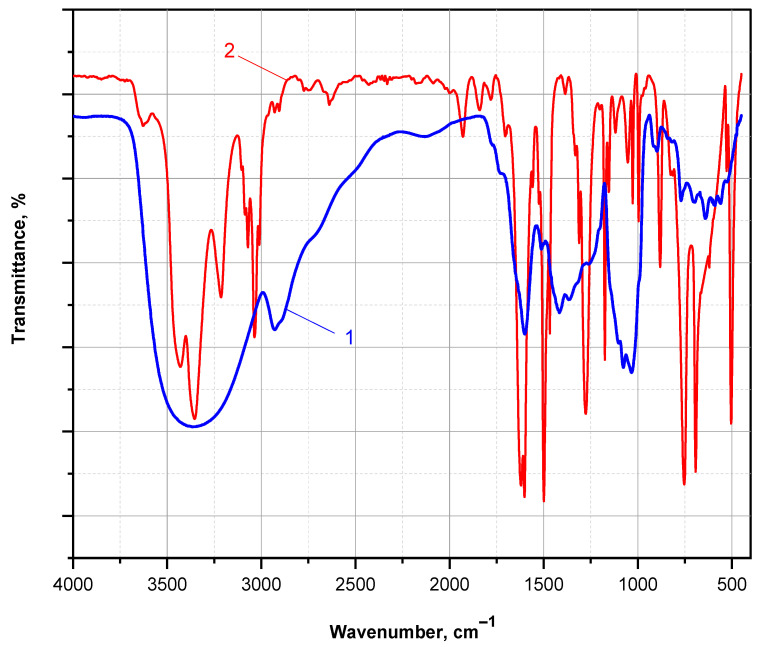
FTIR spectra (KBr pellet, transmission mode) comparing (1) the ANI monomer and (2) the PANI polymer extracted with DMSO from the PA MP carriers, after solvent evaporation under vacuum.

Figure 10 presents the FTIR spectra of a PANI thin film obtained after DMSO extraction from the PA MP supports, followed by solvent evaporation at 90 °C under vacuum. The FTIR spectrum of the aniline monomer is shown for comparison.

The FTIR spectrum of the ANI monomer exhibits all characteristic bands expected for a monosubstituted aromatic amine. These include a split absorption band at 3437 and 3360 cm^−1^, corresponding to N–H stretching vibrations of the associated –NH_2_ group; strong absorptions at 1605 and 1500 cm^−1^, attributed to C=C stretching in the aromatic ring; a distinct band at 1275 cm^−1^, corresponding to C–N stretching in primary amines; and a doublet at 757 and 694 cm^−1^, typical of out-of-plane C–H bending in monosubstituted benzene rings.

In contrast, the FTIR spectrum of the PANI thin film displays distinct features indicative of the emeraldine base form. A broad band centered at 3358 cm^−1^ is attributed to N–H stretching in associated secondary amine groups. A strong, sharp absorption at 1600 cm^−1^ corresponds to C=C stretching in quinoid units, while a broad composite band centered at 1432 cm^−1^ (with shoulders near 1511, 1415, and 1368 cm^−1^) is associated with C=C stretching in disordered benzenoid segments. Additionally, a well-defined band at 1090 cm^−1^ is assigned to the in-plane bending vibration of C–H in the mixed benzenoid/quinoid structure. These spectral assignments are consistent with those reported for nanostructured PANI films prepared on KBr pellets [42].

These observations confirm that the GOx/HRP-mediated polymerization of ANI, catalyzed by PA MP-immobilized enzyme cascades, leads to the formation of a mixed benzenoid–quinoid structure characteristic of PANI in its emeraldine base form. This oxidation state is the only one that can be proton-doped to yield the electrically conductive emeraldine salt. Therefore, these preliminary findings support the potential of the PA-supported GOx/HRP bienzyme cascade as a sustainable and effective platform for the synthesis of electroconductive PANI-coated polyamides.

## 3. Materials and Methods

### 3.1. Materials and Reagents

The ε-caprolactam monomer (AP-Nylon^®^, 99% purity, ECL) was purchased from Brüggemann Chemical (Heilbronn, Germany). The other two lactam monomers, namely, γ-butyrolactam (GBL) and ω-dodecalactam (DDL), both with a purity of 99%, were supplied by Merck Life Science (Algés, Portugal). Before use, all lactams were kept in a vacuum of approximately 10 mmHg for 1 h at 60 °C to eliminate possible traces of moisture. Brüggolen C20^®^ (C20) from Brüggemann Chemical was used as a polymerization activator. The initiator, sodium dicaprolactamato-bis-(2-methoxy-ethoxo)-aluminate (Dilactamate^®^, DL, 85% solution in toluene), was purchased from Katchem (Prague, Czech Republic) and applied without further treatment. Soft, non-insulated iron particles (Fe content > 99.8%), with average diameters of 3–5 μm, were kindly donated by the manufacturer BASF (Ludwigshafen, Germany). GOx from *Aspergillus niger* type VIII and D-(+) glucose (*purum* p.a.) were purchased from Merck/Sigma Aldrich (Lisbon, Portugal). HRP from *Armoracia rusticana* and CH_3_COONa (99%) were purchased from Alfa Aesar (Lancashire, UK). 3,3′,5,5′-Tetramethylbenzidine (TMB, 99%) was purchased from Acros Organic (Porto Salvo, Portugal). Aniline, toluene, xylene, and methanol were all of analytical grade, purchased from Merck (Lisbon, Portugal), and used as received, with the exception of aniline, which was vacuum distilled just before use.

### 3.2. Synthesis of Polyamide Microparticles

Microparticles of PA6, PA4, and PA12 with embedded Fe were synthesized via AAROP, as described in detail previously [43,44,45]. Briefly, for PA6 and PA12, 0.5 or 0.2 mol of the corresponding lactam monomer and 3 wt.% Fe microparticles (relative to the monomer) were dissolved in 100 mL of a toluene/xylene mixture (1:1 *v*/*v*) under nitrogen. The mixture was refluxed for 10–15 min before the addition of 3.0 mol% DL initiator and 1.5 mol% C20 activator. The reaction proceeded for 2 h under constant stirring (∼800 rpm). AAROP for PA6 was conducted at 125–135 °C and for PA12 at 95 °C. For PA4, AAROP was carried out at 40 °C, whereby the GBL (liquid at 40 °C) served as both the monomer and solvent. Additionally, PA6 microparticles without Fe were prepared for reference.

The overall AAROP schemes are shown in Figure S1 (Supplementary Materials). The resulting polyamide microparticles were obtained as white powders (neat PA samples) or gray metallic powders (PA-Fe samples). The PA microparticles were collected by hot-vacuum filtration of the reaction mixture, washed repeatedly with methanol, and dried in a vacuum oven at 60 °C for 30 min. Soxhlet extraction with methanol for 4 h was performed to remove soluble oligomers. Final products were stored in a desiccator.

### 3.3. Characterization Methods

The scanning electron microscopy (SEM) studies were performed in a NanoSEM-200 apparatus of FEI Nova (Hillsboro, OR, USA) using mixed secondary electron/back-scattered electron in-lens detection. All samples were observed after sputter-coating with Au/Pd alloy in the 208 HR equipment of Cressington Scientific Instruments (Watford, UK), with high-resolution thickness control.

UV–VIS absorbance spectral measurements were carried out using a 1900i spectrophotometer from Shimadzu (Kyoto, Japan). The infrared spectra were obtained in a FTIR-4600 apparatus of JASCO (Tokyo, Japan) at room temperature, with a resolution of 4 cm^−1^ accumulating up to 64 spectra for the optimum signal-to-noise ratio.

The real metal load (*R_L_*) of the Fe-containing samples and the thermal stability of all GOx/HRP@PA systems were established by means of thermo-gravimetric analysis (TGA) in a Q500 gravimetric balance (TA Instruments, New Castle, DE, USA), heating the samples in the 40–600 °C range at 20 °C/min in a nitrogen atmosphere. The *R_L_* was calculated according to Equation (1):

(1) $$R_{L}={(R}_{PA\text{-}Fe}-R_{PA})\times 100,[\%]$$

where *R_PA_* is the carbonized residue at 600 °C of the neat PA MP, and *R_PA-Fe_* represents the carbonized residue of the respective Fe-containing PA MP samples.

The average viscosimetric molecular weight (*M_η_*) was determined by intrinsic viscosity measurements in 97% sulfuric acid at a concentration of 0.2 g/dL, using a suspended-level Ubbelohde viscometer thermostated at 25 °C. The calculations were based on the Mark–Houwink equation. For the PA6-based samples, the constants used were *K* = 6.30 × 10^−4^ mL/g and *α* = 0.764 [46]; for PA12-Fe, *K* = 5.58 × 10^−4^ mL/g and *α* = 0.81 [47]. Due to the lack of specific Mark–Houwink parameters for PA4-Fe, the values reported for PA6 were applied.

### 3.4. Adsorption Immobilization of HRP and GOx on PA MPs

Enzyme immobilization was achieved by sequential adsorption. Stock solutions of GOx and HRP (1.0 mg/mL each) were prepared in double-distilled water. To prepare the bienzyme immobilized systems, 50 mg of PA microparticles was suspended in 5 mL of HRP solution and incubated at 37 °C for 8 h under mild agitation. After decanting and storing the supernatant for analysis, the resulting HRP@PA samples were incubated in 5 mL of GOx solution under the same conditions for an additional 16 h. This procedure was optimized based on our previous studies [30,31]. After the final incubation, samples were centrifuged, and the supernatants collected. Immobilized systems designated as GOx/HRP@PA were washed twice with double-distilled water to remove any non-adsorbed enzyme.

### 3.5. Determination of the Total Protein Content in PA6 Microparticles

The amount of immobilized protein was calculated by subtracting the residual protein in the supernatant from the initial protein concentration:

(2) $$T_{P}=C_{0}-C_{S},\;[mg]$$

where *C*_0_ is the initial protein concentration, and *C_S_* is the concentration after immobilization. *C_S_* was determined by direct UV absorbance at ~273 nm, corresponding to aromatic residues (phenylalanine, tryptophan, tyrosine). This method, with a 3–5% standard deviation, offers better precision than colorimetric assays (e.g., Bradford, BCA), which may show up to 15% variability. Quantification was performed using a standard calibration curve [30,31].

### 3.6. Activity Measurements by UV–VIS

To evaluate enzymatic activity, a reaction mixture was prepared by adding 0.1 mL of 0.1 wt.% TMB (in ethanol), 0.015 mL of GOx (1 mg/mL), 0.005 mL of HRP (1 mg/mL), and 0.016 mL of 5 mM glucose to 1.864 mL of acetate buffer (0.1 M, pH 4.5). The reaction was monitored for 1 min at 20 ± 2 °C (room temperature, RT). For immobilized systems, 5 mg of each wet GOx/HRP@PA complex was used instead of native enzymes.

The initial reaction rate was determined from the linear region (0–8 s) of the absorbance vs. time plot. Enzymatic activity was expressed in μkatals, where 1 μkat corresponds to the conversion of 1 μmol of TMB per second under the specified conditions. Specific activity (μkat/mg protein) and relative activity (compared to the GOx/HRP enzyme pair, taken as 100%) were also calculated for comparison.

### 3.7. Cascade Kinetics Studies

Cascade kinetic assays were performed at room temperature in 0.1 M acetate buffer (pH 4.5), with glucose concentrations ranging from 50 to 150 μM in a total volume of 2 mL, including 0.1 mL of TMB solution (0.1 wt.% in ethanol). For the native enzyme dyad, 0.015 mL of GOx and 0.005 mL of HRP (each at 1 mg/mL) were added. For the immobilized GOx/HRP systems, 5.0 mg of the wet complex was used. The increase in absorbance at 652 nm was monitored for 1 min, and the initial reaction rates (V_0_) were calculated from the slope of the linear portion of the Abs_652_ vs. time curves. Lineweaver–Burk double reciprocal plots were then constructed using Equation (3):

(3) $$\frac{1}{V_{0}}=\frac{K_{M}}{V_{max}}\frac{1}{\left[S\right]}+\frac{1}{V_{max}}$$

where *V*_0_ is the initial reaction rate, [*S*] is the glucose concentration, *V_max_* is the maximum velocity, and *K_M_* is the Michaelis constant. *V_max_* values were obtained from the *x*-axis intercepts of the fitted linear plots, and *K_m_* values were subsequently calculated from the slopes.

### 3.8. Enzyme-Mediated Polymerization of Aniline

To evaluate the catalytic activity of the immobilized GOx/HRP@PA complexes in comparison with the GOx/HRP cascade, oxidative polymerization of ANI was conducted at room temperature in 0.1 M acetate buffer (pH = 4.5).

For the reaction using native enzymes, a mixture of 0.15 mL HRP (18.4 µM) and 0.30 mL GOx (2.0 µM) was prepared, to which 0.30 mL of 40 mM ANI was added. Polymerization was initiated by the addition of 1.5 mL of 200 mM D-glucose. This resulted in a final reaction mixture containing 4 mM ANI, 1.0 µM HRP, and 0.2 µM GOx. Formation of PANI occurred rapidly, indicated by a color change from violet to dark brown. After 24 h, PANI precipitated from the solution.

For the reactions involving immobilized enzyme cascades, 50 mg of each wet GOx/HRP@PA complex was used in place of the GOx/HRP enzymes. In these cases, the resulting PANI adhered to the polyamide microparticles, leaving the supernatant nearly colorless, indicating localization of the resulting PANI on the PA MP support.

## 4. Conclusions

This study presents the first attempt to synthesize and characterize cascade biocatalysts based on the GOx/HRP cascade immobilized onto microparticulate supports composed of four different polyamide types, with and without magnetic properties. The supports were synthesized in-house via AAROP under varying conditions, resulting in microparticles with distinct sizes, shapes, and surface morphologies.

The GOx/HRP enzyme cascade was noncovalently immobilized onto the polyamide supports through sequential adsorption, with precise control over the amount of immobilized protein. Activity studies showed that the PA4-Fe-supported dyad exhibited over 50% higher activity than the native GOx/HRP system, while the PA6-supported complex demonstrated comparable but slightly lower performance.

For kinetic evaluation, the widely used single-substrate Michaelis–Menten model was applied to all GOx/HRP systems. This approach was justified by the constant presence of glucose in large excess, resulting in reaction kinetics being effectively governed by the HRP-mediated oxidation of aniline, with H_2_O_2_ acting as an intermediate. Under these conditions, the cascade operates in a pseudo-steady-state regime, allowing the extraction of apparent Vₘₐₓ and Kₘ values. Their kinetic analysis revealed that all immobilized systems—except for that based on PA12-Fe—achieved catalytic efficiencies comparable to or exceeding those of the native enzyme cascade.

The ability of the novel PA MP-immobilized systems to catalyze the polymerization of ANI was further confirmed by UV–VIS and FTIR analyses that showed the predominant formation of PANI in its emeraldine base form, even in the absence of template molecules. To the best of our knowledge, this is the first attempt to polymerize ANI with an immobilized GOx/HRP bienzyme cascade.

These findings support the feasibility of using polyamide microparticle-supported enzymatic systems for the synthesis of PANI. The approach holds promise for the development of layered polymeric materials incorporating conductive PANI coatings, with potential applications in flexible electronics and other advanced technologies.

## Figures and Tables

**Table 1:** Some basic characteristics of the synthesized microparticulate polyamide supports.

Sample	Yield of Polymer [%]	Oligomers ^1^ [wt.%]	*M_η_* [kDa] ^2^	Average Particle Size [µm]	Average Pore Size ^3^ [nm]	Fe Content ^4^ [wt.%]
PA4-Fe	61.2	5.0	18.0	10–30	120–300	1.0 [1.9]
PA6	56.0	2.0	29.7	100–200	100–1000	-
PA6-Fe	58.8	2.5	22.8	30–150	100–1000	3.0 [5.6]
PA12-Fe	58.5	3.0	25.0	20–100	200–800	2.0 [4.8]

^1^ After Soxhlet extraction; ^2^ Viscosity average molecular weight *M_η_* determined in H_2_SO_4_ (for details, see Section 3.3); ^3^ Particle and pore sizes were determined from SEM micrographs by means of the respective tools of the SEM software (xT control server, version 1.3.3.); ^4^ In relation to monomer before polymerization and in the final polymer (in brackets) determined by thermogravimetry according to Equation (1) (Section 3.3).

**Table 2:** Activity parameters of the immobilized GOx/HRP@PA systems compared to GOx/HRP.

Sample	Total Protein [mg]	Slope [Abs_652_ s^−1^]	Specific Activity [µkat.mg^−1^]	Relative Activity [%]
Native GOx/HRP	0.02000	0.1785	4.577	100.0
GOx/HRP@PA6	0.00862	0.0447	1.147	25.1
GOx/HRP@PA6-Fe	0.00845	0.0988	2.533	55.3
GOx/HRP@PA4-Fe	0.00206	0.2767	7.094	155.0
GOx/HRP@PA12-Fe	0.01195	0.0182	0.467	10.2

Note: Results are averaged over three independent activity assays; the standard deviation ranges between 3% and 5%. TMB extinction coefficient ε = 39,000 M^−1^cm^−1^.

**Table 3:** Kinetic parameters for all GOx/HRP cascades derived from the double reciprocal plots.

System Designation	*K_m_* [µM]	*V_max_* [µM s^−1^·mg^−1^]	CE × 10^2^ [s^−1^]
GOx/HRP	275	11.6	4.22
GOx/HRP@PA6-Fe	397	20.1	5.06
GOx/HRP@PA4-Fe	1643	73.9	4.50
GOx/HRP@PA12-Fe	1286	13.3	1.03
GOx/HRP@PA6	254	11.0	4.34

## Data Availability

All the data generated during this research are presented in the manuscript and in the Supplementary Materials.

## Supplementary Materials

The following supporting information can be downloaded at: https://www.mdpi.com/article/10.3390/molecules30143003/s1: Figure S1: Chemical structures of the reagents participating in AAROP and of the microparticulate polyamides prepared; Figure S2: Schematic representation of the bienzyme cascade reaction used for β-D-glucose colorimetric determination according to earlier studies; Figure S3: Double reciprocal Lineweaver—Burk plots for the native enzymes and the GOx/HRP@PA immobilized cascades; Figure S4: Representative structures of PANI in its three oxidation stages.

## Abbreviations

The following important abbreviations are used in this manuscript:

ANI	Aniline
PANI	Polyaniline
GOx	Glucose oxidase
HRP	Horseradish peroxidase
PA MPs	Polyamide microparticles
PA4	Polyamide 4
PA6	Polyamide 6
TMB	3,3′,5,5′-tetramethylbenzidine
PA12	Polyamide 12
ATR	Attenuated Total Reflection
SEM	Scanning electron microscopy
TGA	Thermogravimetric analysis
FTIR	Infrared spectroscopy with Fourier transform
RT	Room temperature (21 ± 2) °C
AAROP	Activated anionic ring-opening polymerization
GOx/HRP@PA	Generic name of immobilized GOx/HRP cascades
