# Supplementary material for: Noncovalently Immobilized Glucose Oxidase/Horseradish Peroxidase Cascade on Polyamide Supports for Eco-Friendly Polyaniline Synthesis
Source: Molecules. 2025 Jul 17;30(14):3003. doi: 10.3390/molecules30143003 (PMC12300839; doi:10.3390/molecules30143003)
Supplement: Supplementary file 1 [file molecules-30-03003-s001.zip › molecules-3709868-supplementary.pdf]

molecules

MDPI

Supplementary data for review

Noncovalently Immobilized Glucose Oxidase/Horseradish Peroxidase Cascade on Polyamide Supports for Eco-friendly Polyaniline Synthesis

Nadya V. Dencheva*, Joana F. Braz, Sofia A. Guimarães, Zlatan Z. Denchev*

[1] IPC-Institute for Polymers and Composites, University of Minho, Guimarães 4800-056, Portugal; nadiad@dep.uminho.pt; joanabraz@dep.uminho.pt; a101413@alunos.uminho.pt; denchev@dep.uminho.pt

* Correspondence: nadiad@dep.uminho.pt; denchev@dep.uminho.pt

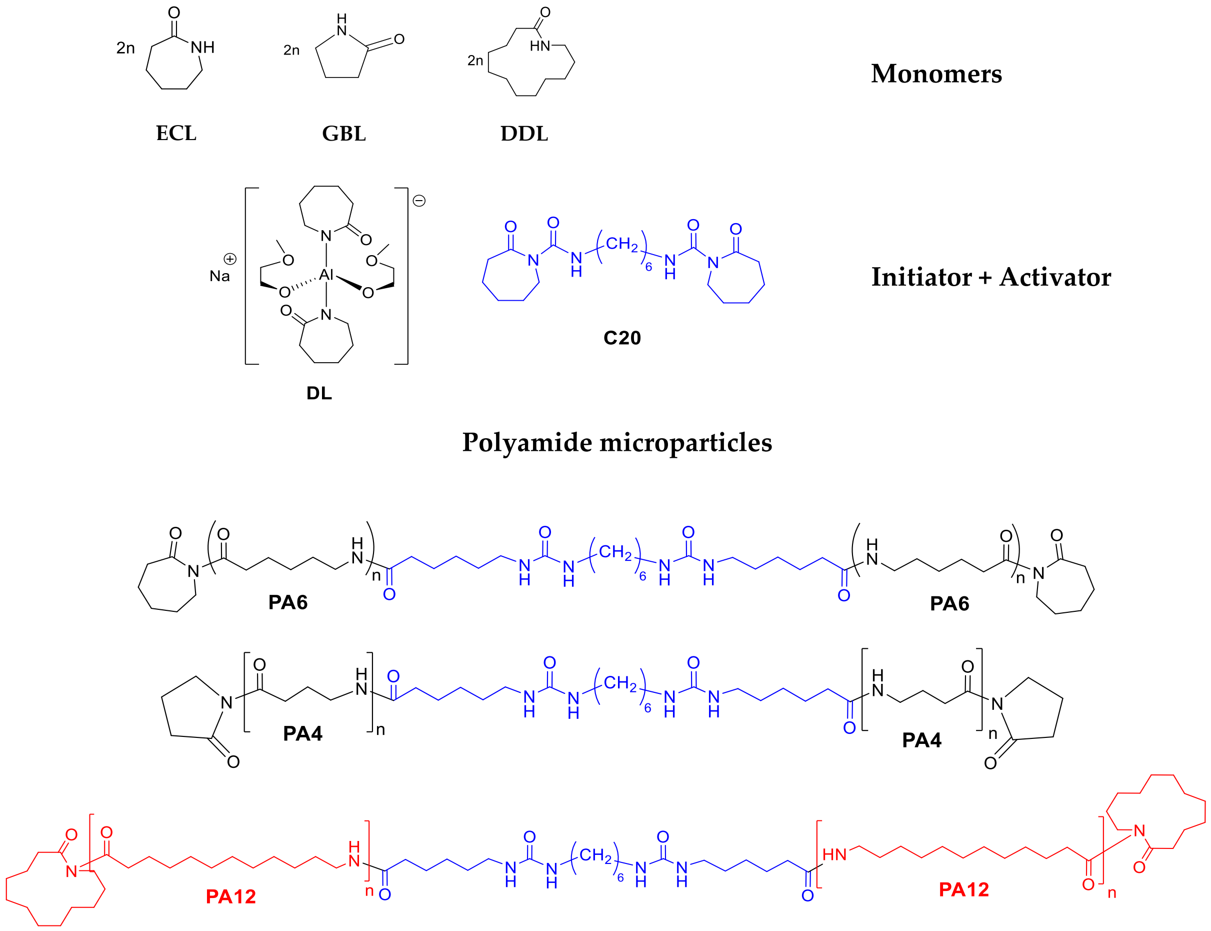

Figure S1. Chemical structures of the reagents participating in AAROP and of the microparticulate polyamides prepared. ECL = ε-caprolactam, the monomer for PA6; GBL = γ-butyrolactam, the monomer for PA4; DDL = ω-dodecalactam, the monomer for PA12. C20 = the active substance of the AAROP activator; DL = dicaprolactamato-bis-(2-methoxyethoxo)-aluminate wherein R = $OCH_2CH2OCH_3$, the anionic initiator of AAROP.

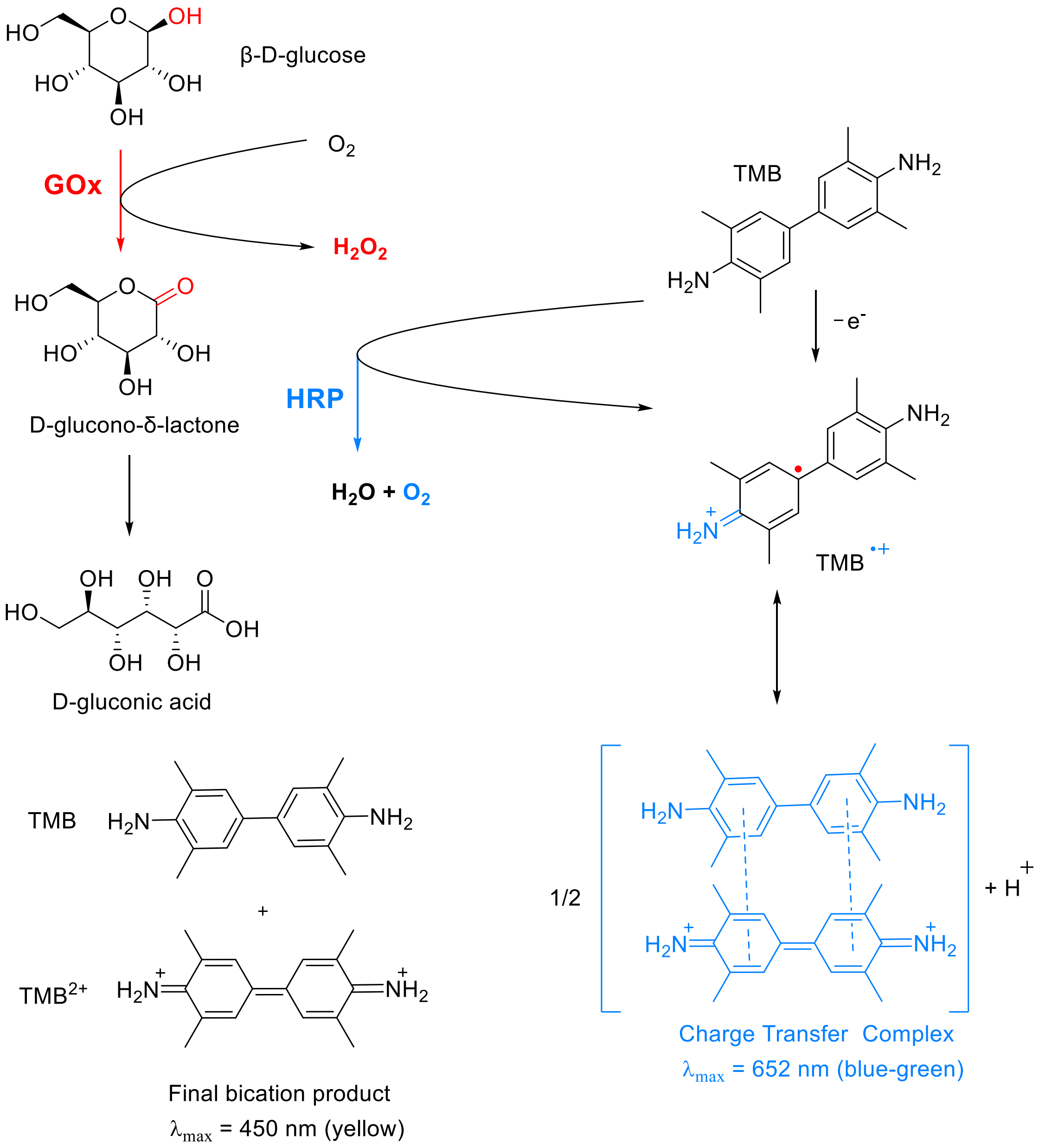

Figure S2. Schematic representation of the bienzyme cascade reaction used for β-D-glucose colorimetric determination according to [1,2]. GOx = glucose oxidase; HRP = horseradish peroxidase; TMB = 3,3′,5,5′-tetramethlbenzidine. [1,2]

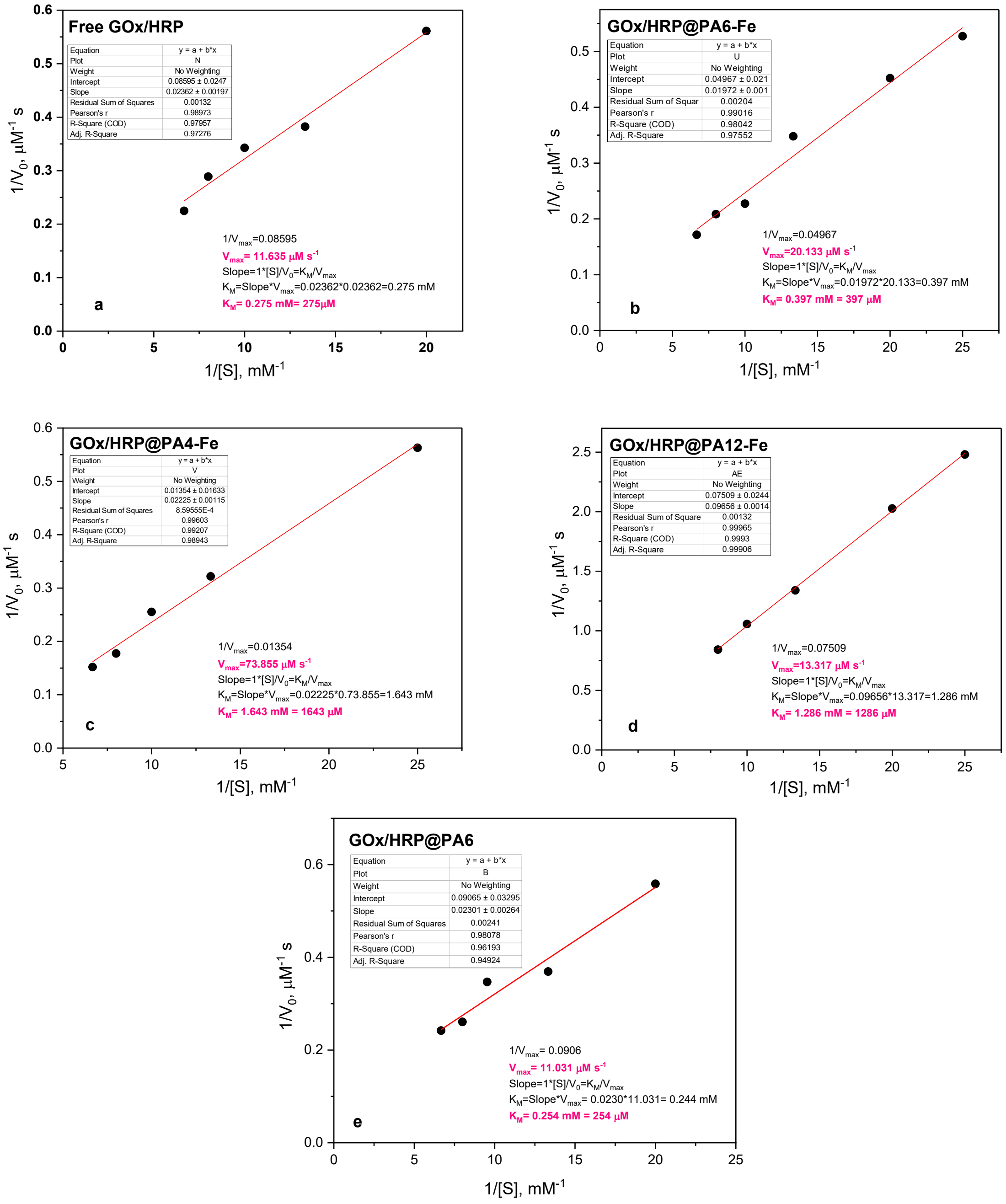

Figure S3. Double reciprocal Lineweaver–Burk plots according to Equation 3 for the free enzymes and the GOx/HRP@PA immobilized cascades: a – free GOx/HRP; b – GOx/HRP@PA6-Fe hybrid system; c -– GOx/HRP@PA4-Fe; d – GOx/HRP@PA12-Fe; d – GOx/HRP@PA6. GOx = glucose oxidase; HRP = horseradish peroxidase.

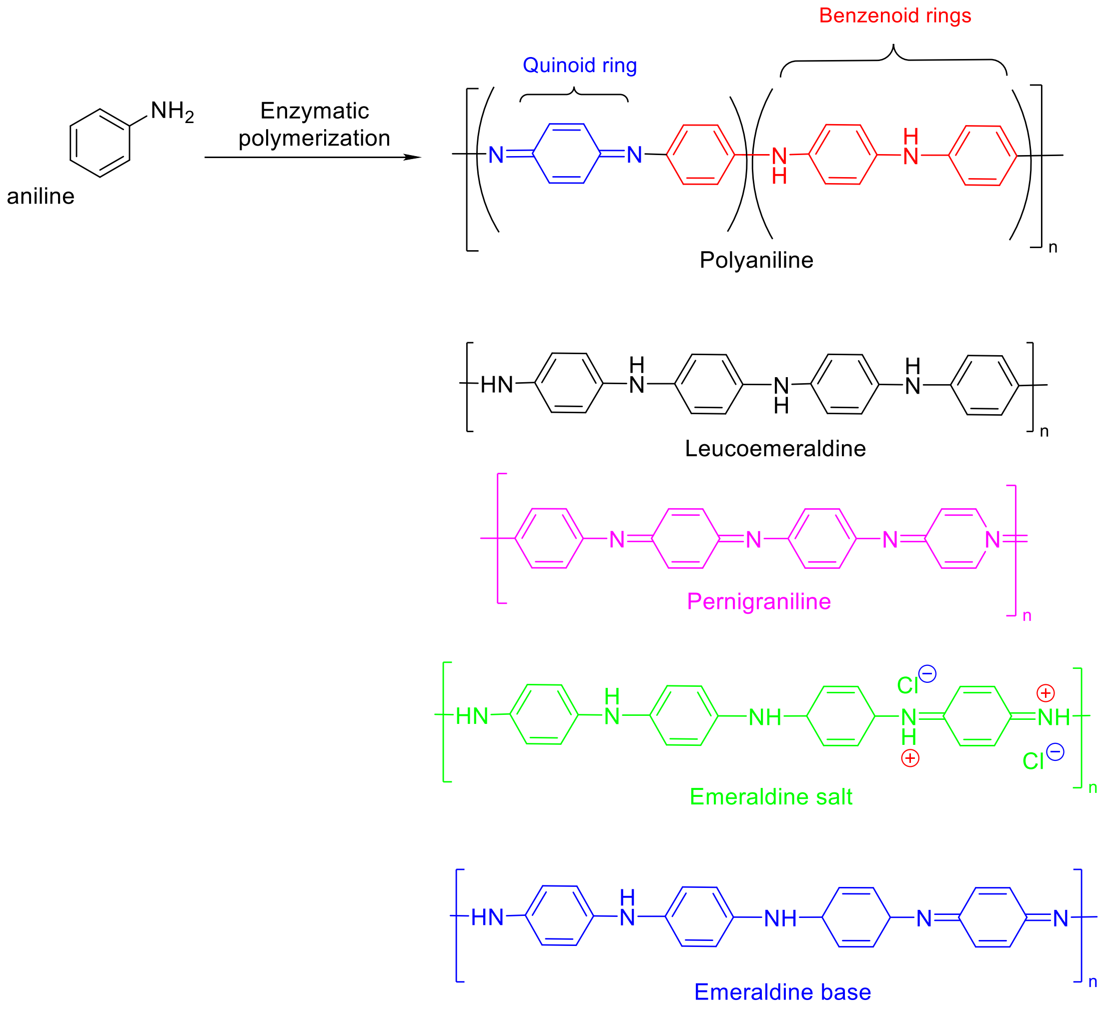

Figure S4. Representative chemical structures of polyaniline (PANI) in its various oxidation states, distinguished by the relative content and arrangement of benzenoid and quinoid rings. *Leucoemeraldine base* consists entirely of benzenoid rings and is electrically insulating. *Pernigraniline base*, also an insulator, represents the fully oxidized form of PANI and is composed exclusively of quinoid structures. *Emeraldine base* contains an equal ratio of benzenoid and quinoid units and is the only oxidation state that can be protonated (doped) to form the electrically conductive *emeraldine salt*.

References:

1. Josephy, P.D.; Eling, T.; Mason, R.P. The horseradish peroxidase-catalyzed oxidation of 3,5,3′,5′-tetramethylbenzidine. Free radical and charge-transfer complex intermediates. *J. Biol. Chem.* **1982**, *257*, 3669–3675.

2. Misono, Y.; Ohkata, Y.; Morikawa, T.; Itoh, K. Resonance Raman and absorption spectroscopic studies on the electrochemical oxidation processes of 3,3′,5,5′-tetramethylbenzidine. *J. Electroanal. Chem.* **1997**, *436*, 203–212.
